# Psychosocial Experiences That Support Positive Self-Concept in Children with Cleft Lip and Palate Adopted From China

**DOI:** 10.1177/10556656211051222

**Published:** 2021-12-03

**Authors:** S. A. Everhart, K. M. Cochran, N. H. Lambrou, W. H. Davies, P. K. Marik

**Affiliations:** 1 20209 Children’s Hospital of Orange County, Orange, CA, USA; 2 14751 University of Wisconsin-Milwaukee, Milwaukee, WI, USA; 3 Lighthouse Clinic, LLC, Milwaukee, WI, USA; 4 Geriatric Research Education and Clinical Center, 20132 Wm S Middleton Memorial Veterans Hospital, Madison, WI, USA; 5 School of Medicine and Public Health, University of Wisconsin-Madison, Madison, WI, USA; 6 5497 Children’s Wisconsin, Milwaukee, WI, USA; 7 5506 Medical College of Wisconsin, Milwaukee, WI, USA

**Keywords:** cleft lip and palate, international adoption, self-concept, psychosocial adjustment, cultural identity

## Abstract

**Objective:**

Existing psychosocial research offers little information on the unique challenges and strengths of children adopted from China with cleft lip and/or palate (CL/P). The present study aimed to understand biopsychosocial factors that support positive self-concept in this population.

**Design:**

Qualitative, semistructured interviews were conducted with children and their parents. Interpretive phenomenological analysis of transcribed interviews was utilized for data analysis.

**Setting:**

Participants were recruited in an outpatient, pediatric multidisciplinary cleft clinic during a standard team visit.

**Patients, Participants:**

Participants were ages 8 to 12 years with a diagnosis of isolated cleft lip-palate who were internationally adopted from China before the age of 2 years and English-speaking. Participants also included English-speaking parents.

**Results:**

Themes reflecting data from the child and parent subsamples include: (1) child's characteristics, (2) family strengths, (3) adoption identity, (4) cultural identity, (5) coping with a cleft, and (6) social factors. Additional 2 to 3 subthemes were identified for the parent and child subsamples within each broader theme.

**Conclusions:**

Findings from this sample suggested factors supporting positive self-concept included pride and self-efficacy in activities, family support, instilment of family values, strategies for coping with a cleft, family belonging, cultural exposure, and normalization of differences. Medical providers can support patients and families by providing education on surgeries, CL/P sequelae and outcomes, and pediatric medical stress. Mental health providers can screen for social and emotional challenges and provide psychoeducation on racial/ethnic socialization, identity development, and coping.

## Introduction

It is estimated that 1 in 500 births in China occurs with cleft lip and/or palate (CL/P). Because China is one of the most populous countries in the world, this results in a high prevalence of individuals with CL/P of Chinese descent (Kling et al., 2014). Within the United States, it is common for children with CL/P to have been adopted from China and other East Asian countries (Hansson et al., 2012). Research has documented poorer medical outcomes for children with CL/P adopted from China, including receipt of initial repair surgeries later than recommended (Werker et al., 2017; Kaye et al., 2019) and greater likelihood of surgical revisions and higher fistula rate (Swanson et al., 2014), though some research has suggested similar rates of revisions and fistulas for those adopted children compared to nonadopted children (Goldstein et al., 2014). Psychosocially, even mild preadoption adversity predicts internalizing and externalizing problems (Tan and Marfo, 2016). In a review of international adoption research trends, Palacios and Brodzinsky (2010) observed a greater likelihood of adoptees being referred for mental health treatment, exhibiting psychological problems, and performing poorer in school compared to nonadoptees.

Research has highlighted the role of self-appraisal, self-concept, and other self-perceptions in promoting positive psychosocial outcomes for those with CL/P (Rumsey and Harcourt, 2005; Feragen et al., 2009). For example, the authors of 1 study found that 10-year-old children with CL/P classified to be psychosocially resilient reported less teasing, staring, and questions compared to those in the nonresilient group. Perceptions of teasing had a greater association with resilience than cleft visibility or parents' satisfaction with child appearance or speech (Feragen et al., 2009). A review article authored by Richman et al. (2012) implicated the importance of satisfaction with appearance over variables such as type of cleft, age, and gender in predicting positive psychosocial outcomes. Previous findings regarding the self-concept of children with CL/P have been mixed with some suggesting lower self-concept scores relative to peers (Kapp-Simon, 1986), with others indicating average to above average scores (Leonard et al., 1991; Persson et al., 2009).

In the broader child and development literature, self-concept has been identified as an important aspect of social and emotional development (Harter, 2006). Development of self-concept in middle to late childhood includes growing self-awareness, greater emphasis on the self in relation to others, and a more global self-esteem compared to earlier childhood years (Harter, 2012). Normative cognitive development tends to have predictable impacts on self-concept development, while social interactions and cultural context are likely to contribute to individual differences (Harter, 2012). Temperament and family interactions have been shown to influence the development of self-concept in children (Thompson, 1998; Brown et al., 2009) with positive interactions with parents serving as a buffer against a child's proneness-to-distress (Brown et al., 2009). Self-esteem, an aggregated view of the self which includes evaluations of the self (Rosenberg, 1979), has also been linked with a variety of psychosocial outcomes. Low self-esteem in adolescence has been associated with depression in adulthood (Sowislo and Orth, 2013; Steiger et al., 2014), poorer mental and physical health, and higher levels of criminal behavior compared to those with high self-esteem in adolescence (Trzesniewski et al., 2006). On the other hand, high self-esteem has been associated with positive affect, relationship satisfaction, job satisfaction, and lower rates of depression (Orth and Robins, 2013).

While there are studies related to medical, speech, and language outcomes for internationally adopted children, few describe psychological and social outcomes for internationally adopted children with CL/P. To date, a critical gap in the literature exists when comparing internationally adopted to nonadopted children with CL/P, studies examining self-concept, and studies examining psychological adjustment in this population. Existing studies examining psychosocial outcomes of children with a cleft generally have not specified if samples included internationally adopted children. Thus, the present study utilized qualitative methods to explore experiences of school-age children with CL/P adopted from China within biopsychosocial and phenomenological frames. Phenomenology centers on the experiences of the participants, which was a key aim of the present study. Self-concept was chosen as a guiding construct for the present research study because of its prominence in the literature for nonadopted children with CL/P (see Stock and Feragen, 2016).

## Methods

### Participants

Participants were eligible for participation in the study if they had a diagnosis of CL/P, were between the ages of 8 and 12 years, were internationally adopted from China, and spoke English. Exclusionary factors included unwillingness of children or parents to participate; limited English proficiency for either child or parent; active suicidal ideation or other acute mental health concern; and cognitive or developmental delay limiting ability to freely give consent/assent or understand interview questions. Fourteen child–parent dyads were approached for participation in the present study. Of those, 1 participant was ineligible due to suicidal ideation. Two declined in person. Two agreed to participate later by phone but were not able to be reached. Participants were dyads (*n* = 9) of parents and children ages 8 to 12 years (mean age = 9.8 years, SD = 1.9 years) with a diagnosis of isolated cleft lip–palate (CLP) and who were adopted before the age of 2 years. The child subsample included 6 girls and 3 boys. Five families endorsed speech and/or hearing concerns. The presence of speech and/or hearing concerns was coded as present if either the child or parent raised the subject during the course of the interview (*n* = 5), or if participants had low intelligibility (*n* = 2). This was measured during the data analysis when transcripts included multiple quotes from child participants that were indistinct to the primary researcher. Parents more often discussed speech/hearing concerns, while children did not explicitly mention speech/hearing unless prompted to by a parent. This may be an underestimation of the true rate of speech/hearing concerns in the sample due to the high frequency of such problems for the pediatric CL/P population; although specific prevalence rates for speech and/or hearing deficits are difficult to find for school-age children, studies have documented high rates of speech concerns for those with UCLP (see Lohmander and Persson, 2008) and in those with cleft palate ± lip (Prathanee et al., 2013). Finally, about half of the sample endorsed social and/or emotional concerns (*n* = 4), again measured by the child and parent report during the course of interviews. Parents were predominantly White (*n* = 9) and 1 parent was Asian (*n* = 1). See Table 1 for complete demographics.

**Table 1.** Sample Demographics.

Age	*M* = 9.88	SD = 1.19
Gender		
Female	6	67%
Male	3	33%
Diagnosis		
CLP	9	100%
Comorbidities		
Speech	5^a^	56%
Hearing	4^b^	44%
Social/emotional	4^b^	44%
Parents		
Mother	9	89%
Mother and father	1	11%

^a^ Parent-reported or low intelligibility during interviews.

^b^ Parent-reported during interviews.

### Data Collection and Analysis

Participants were recruited from a multidisciplinary craniofacial and CL/P clinic during a standard team visit. Qualitative data were collected from children and their parents through in-person semistructured interviews conducted by 1 of 2 graduate students. Interviewers asked participants a predetermined list of questions developed for child and participant subsamples (see Supplemental online appendix). Interviewers asked follow-up questions as needed to elicit participant elaboration. Child and parent interviews were typically conducted separately though participants were given the option of having the other family member present if they preferred. About one-third of dyads elected to complete interviews together. Interviews were completed after the final question from the interview guide was asked, and there was no time limit. Child interviews lasted between 18 and 30 min while parent interviews ranged from 28 to 45 min. Interviews were audio-recorded, transcribed verbatim, deidentified, and then analyzed with interpretive phenomenological analysis (IPA; Smith, 1996). IPA is an inductive process that seeks to ensure the validity, reliability, and integrity of the research. IPA was chosen for analysis due to its emphasis on phenomenology, the lived experiences of participants, and its established use in the field of health psychology. Studies utilizing IPA have focused on illness experience, carers' experiences, and health professionals' experience, among others (Smith, 2011). The primary researcher and 2 additional doctoral-level researchers assisted in qualitative coding and theming. Participation of multiple coders allows for bias-checking, collaboration, and consensus processes. After familiarizing themselves with the data, coders initially coded the transcripts, which consisted of simple line-by-line descriptor labels of the data.

Next, emergent theme development sought to include the interpretation of the researchers while also maintaining an accurate representation of participant quotes. In contrast to the looseness and openness of open codes, emergent themes were developed to succinctly reflect an understanding (Smith et al., 2009). For both initial coding and emergent theme development, deidentified transcripts were distributed evenly amongst the 3 coders. Child and parent dyads were separated so that 1 coder did not code both the child and parent of any 1 dyad at a particular level of analysis. Following independent development of emergent themes, the primary researcher edited and consolidated the list of themes for both children and parent subsamples separately. Emergent themes were retained if they arose in at least one-third of interviews. This list of emergent themes was then reviewed by other coders to ensure accurate characterization of researcher interpretations.

**Figure 1.**
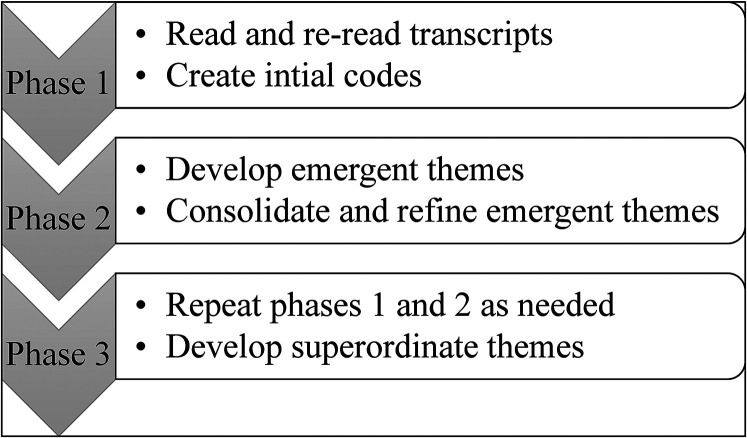
IPA coding procedures.

^a^Adapted from Smith et al. (2009).

Following the development of emergent themes, researchers collaboratively developed superordinate themes utilizing abstraction (clustering themes and labeling the group with a new superordinate theme), subsumption (when an emergent theme became superordinate and brought together other related emergent themes), and polarization (pulling together opposing themes). See Figure 1 for IPA coding procedures.

## Results

Superordinate themes reflecting data from the child and parent subsamples include (1) child's characteristics, (2) family strengths, (3) adoption identity, (4) cultural identity, (5) coping with a cleft, and (6) social factors. Each subsample had unique emergent themes though some were common to both subsamples (see Table 2).

**Table 2.** Superordinate Themes and Child and Parent Emergent Themes.

Superordinate Themes	Emergent Themes	
	Child	Parent
Child's characteristics	Pride in extra-curricular/interests	Child has many positive qualities
	Positive attributes and preferences	Child is resilient and adaptive
	Appearance perceptions	
Family strengths	Safety and validation	Togetherness and support
	Parents instill and reinforce pride	Family values
		Talking through situations helps a child cope
Adoption identity	Pride in adoption	Social support while adopting
	Open and honest conversations	Impact of developmental trauma
	Tangible mementos	
Cultural identity	Understanding of the word “culture"	Lack of perceived discrimination toward child
	Limited contact with others from China, adopted, or with cleft	Incorporating Chinese culture
		Cultural exposure is child-led
Coping with cleft	Cleft associated with surgeries	Transparent conversations normalize appearance differences
	Cleft is just part of life	Cleft is not a big deal, but is still chronic
Social factors	Only discussed adoption with trusted persons	Social skills challenges
	Perceptions of bullying/teasing	Speech and hearing deficits increase the risk

### Child's Characteristics

#### Child

When asked to describe themselves, participants often replied with their activity involvement, typically endorsing *pride and self-efficacy in extra-curricular activities and interests.* For example, 1 participant reported they are really good at “singing and doing gymnastics.” One participant reported they “like to play basketball and soccer. [They] like dance and [they] like art and hanging with [their] friends.”

When asked about individual characteristics, child participants overwhelmingly described *positive attributes*. When asked to share facets they liked and disliked about their appearance, child participants typically reported neutral or positive overall *appearance perceptions.* One participant shared, “I like [my appearance]. I like my new glasses. I like my thick hair. I like my dimples.” Three participants described negative appearance perceptions regarding orofacial features. The first noted that his smile is “weird” and “disgusting.” A second participant said, “I don't like how I have a flat nose. You know what I’m saying? It's different but also unique.”

#### Parent

Just as children described themselves in terms of positive attributes, parents, too, emphasized the *positive qualities* of their children. Children were described as “lovable,” “caring,” “an angel,” “outgoing,” “sweet”, “helpful,” “awesome big sister,” “creative,” “compassionate,” and “polite.” With these positive qualities was also parent awareness of child sensitivity. For example, 1 parent described her child as “… very sensitive, kind-hearted. And on the other opposite end of the spectrum, she is very stubborn and very fiery… And is hurt very easily.” Another strength noted by the parent subsample was the *resilience and adaptiveness* of their children. For example, 1 parent participant described adaptations her child made for speech challenges: “Well because communication is difficult at first, so we adopted her at age 2 and she couldn't ask what she want, what she can do, and so, she have to [*sic*] figure things out on her own.”

### Family Strengths

#### Child

Child participants generally described their parents and/or family to be their primary source of support. *Safety and validation* captured the sense of consistent presence, comfort, and support provided by parents. For instance, 1 participant simply stated parents help by “making me less scared.” This emergent theme was evident, too, when children shared advice that they would give to other children who were adopted. For example, 1 participant stated, “no matter what, their families [*sic*] is going to take care of you. That's why they adopted you, because they wanted another kid, or they wanted a kid.”

The final emergent theme demonstrative of the family as a primary source of support was the ways in which *parents instilled and reinforced pride* in their children. This was evident for child–parent dyads where the parent was in the room during the child interview. For instance, 1 child participant was having a difficult time identifying something of which they were personally proud. The mother in the room then offered, “What about when you earned your black dress?” At that, the child participant replied, “I was happy and excited.”

#### Parent

It was evident across the subsample that families emphasized *togetherness and support.* One example of family supporting coping was when 1 mother talked about being there for a surgery: “Well, I guess just reassuring him that we’re going to be there when he gets out. That's all you can do.” One mother said, “I think we’re very close. Very supportive … spending time together. Um, being there for each other. Yeah, that's pretty much, I mean, we’re, we’re blessed [*sic*].”

In addition to the togetherness described by parent participants*, family values,* often including religious faith, were discussed. One participant described values of respect and helpfulness: “Being respectful of each other. Being helpful … everybody's got their responsibilities in the family.” In addition to family values, parent participants highlighted the importance of *talking through situations to help their children cope.* One mother described helping her child:

And she and she got [*sic*] kind of emotionally attached to the situation where we had to talk her through and say, “actually this is a very unhealthy situation” … So, we had to just talk her through that and just explain it.

### Adoption Identity

#### Child

For all participants, adoption occurred prior to the age of 2 years old, meaning the children themselves could not remember the adoption. However, child participants often knew about the details of their adoption story, such as who was there to pick them up in China, specific trip elements, and who was waiting to greet them at home. Similarly, child participants articulated *pride and positive perceptions of their adoption*. One participant stated, “… and I also feel happy about the adoption that we had. That is God moment [*sic*] right there.”

Child participants described how they and their families had *open and honest conversations about adoption*. This was apparent in the comfort participants demonstrated in discussing adoption with the interviewer as well as the content of their replies. Contrasted with questions about cleft and appearance perceptions, children typically had more to say regarding their adoption story. When asked when they first learned the story of their adoption, 1 participant said, “I don't know. Maybe like five or something?” The participant's mother elaborated:

We’ve always talked about it. We have a binder of some pictures of when he was in the orphanage … the binder with the white cat. [to child] Remember you have the pictures of you in the crib and then you with your other friends from the orphanage that were in the walkers—we have pictures of that. So, we’ve always been very open. We’ve never not talked about it.

The quote above illustrates an additional theme of *tangible mementos.* Children, or parents during the child interview, often discussed a photo album or book used to commemorate the story of their adoption.

#### Parent

Each parent described how they were inspired to adopt. This meant a thoughtful decision-making process and typically years of waiting. *Social support while adopting* was described as helpful for families. It was common for families to learn about the adoption process or take classes with others, and to travel to China with other adopting families. Social support was also helpful for some participants who spoke with others about CL/P prior to the adoption.

*The impact of developmental trauma* was highlighted by parents to whom it was relevant. Three parent participants endorsed experiences of trauma sequelae including attachment concerns, regression, disruptive behavior, or social/emotional difficulties. One parent participant described CLP as secondary to issues related to developmental trauma, stating, “yeah we had a lot of developmental issues … Lots of neglect that we weren't prepared for prior to the adoption.”

Just as child participants highlighted the pattern of open and honest conversations about adoption, parent participants also verbalized ways in which *regular conversations normalize adoption.* One participant stated, “… we’ve always been open with her adoption and her CLP right from the beginning.” One parent described the dialogue around racial differences: “We talk about the difference of the way that he looks and the way that I look. And that's because he's Chinese. Yeah, and so sometimes we’ll talk about his eyes, his almond shaped eyes, right?” One parent reported talking about adoption as their child ages: “So, now obviously as she grows older, you know there's questions of why she doesn't look like us and why doesn't … you know, just the whole adoption story. We’ve always tried to keep that in front of her.”

### Cultural Identity

#### Child

When asked about their *understanding of the word* “culture,” most indicated a basic understanding of the construct, while 2 indicated good understanding. One participant shared the following:

I know that they have a different language, culture, silk—but we do have silk, but from China… and I also learned that my aunt has been there and that when you hear the language—it's so beautiful. It's so beautiful … I think that that means like the way they do [*sic*] … you that the way they live, like culture, our culture … I know that they have letters, Chinese culture, and characters and sounds.

Child participants often had concrete knowledge of China. For instance, 1 participant mentioned, “most things are made there.” Several of the participants identified awareness of the Great Wall of China.

Although some participants endorsed having friends who were also adopted or knowing children or adults from China, participants typically did not know many others who shared similar identity facets such as being adopted, being Chinese, or having CL/P. Overall, child participants had limited contact with others from China, adopted or with a cleft.

#### Parent

Parent participants denied discrimination toward their children. This was often contextualized in the diversity of the community, with both racially homogenous and heterogeneous communities identified as contributing factors. When asked about any bullying or discrimination, 1 parent participant simply stated, “not that I have seen. Not that she's ever told me about. It's a pretty open community.” Another participant reported not having seen discrimination and then reporting, “we’re not completely diverse in [small city], but there's enough diversity even in her school ….” On the other hand, 1 participant identified the value of a diverse community: “As far as Chinese…but we have enough in the community of Asian and African American and Spanish … no one is really like, ‘oh you’re different’. And there's other adoptees from China in her school.”

Parent participants discussed the incorporation of Chinese culture into their lives with artifacts around the home, scrapbooks, photos, or celebrating Lunar New Year. One participant said, “… we try in different ways to do what we can to incorporate small touches. So, she knows that we understand where she's from.” An illustrative remark was offered by another parent: “So, you know, so some simple things. But it's not like we’re totally immersed in it either.” One parent highlighted the challenges in White parents providing cultural exposures outside of their own background, reporting, “yeah. I think it's hard because I feel badly that she doesn't have a lot of memory of her beginning in China. You know, for her, she's very Americanized.” Parent participants described cultural exposures to be child-led. One participant stated, “we might wait until she is a little bit older and maybe has more of a need to learn the culture or want to learn the culture instead of just say ‘here you go.’”

### Coping with Cleft

#### Child

When asked about CLP, it was typically associated with surgeries. It was typical for the child subsample to have clear memories of bone graft surgery given their age and the typical timing of the surgery. When asked what 1 participant knew about CL/P, they simply replied, “I’ve had a lot of surgeries on it.” Another mother reported her child has had “12 to 15 surgeries” including those related to CLP and hearing (ie, ear tubes).

*Cleft is just part of life* is an emergent theme that captured resilience, represented in how participants discussed positive coping with CLP. It was also reflected in the absence of negative verbalizations about cleft. For example, a participant was asked how having a cleft affected life now. The participant responded with a lengthy pause. The interviewer then suggested, “and maybe it doesn't [affect your life now].” To this, the participant responded, “yeah, it doesn't.” Another participant stated they “don't really pay attention” to CLP.

Child participants articulated optimism about cleft outcomes. This was evident when participants gave advice to other hypothetical children with CL/P, often stating surgeries will make things better. For example, 1 participant reported they would tell another child, “It's okay; they’re not going to hurt you. And that your mom or your dad is going to be there with you and that you might have no school.”

#### Parent

Similar to conversations surrounding adoption, parent participants also endorsed open dialogue with regard to CLP. These conversations appeared to be a useful strategy for normalizing appearance differences related to CLP. Similarly, these conversations were often described as a matter of fact, giving simple education to children to explain facial differences. Another participant reported responding to the child's questions about why her lip was different with **“**ya [*sic*] know, your lips looks fine, sweetie. It's because of surgery and your face changes and grows and develops as you get older. So, don't worry about it.”

*Cleft is not a big deal but is still chronic* summarizes the duality of CLP both as a burdensome, chronic condition yet manageable. One parent participant stated, “we try to keep it simple,” when describing their approach to managing cleft. Another participant captured the opposition:

So, it's not like heart damage and … it's not like an ongoing chronic condition, but it is… And so, I have gone back to the [adoption] agency and said, “I think that by stating it this way, you’re doing families a disservice”. ‘Cause [*sic*] this is, this has been very much ongoing.

### Social Factors

#### Child

When asked about sharing adoption stories with peers or answering questions about race or background, child participants typically reported they *only discussed adoption with trusted persons*. One participant reported she would only share information with best friends.

When asked about their *perceptions of bullying/teasing*, child participants most often reported some experiences of teasing or bullying (*n* = 6). Few denied experiences of teasing or bullying (*n* = 3). Notably, for those that did endorse teasing/bullying, interactions were described to be relatively minor by both children and parents. One participant reported being bullied due to her size. Another participant described their “brother picking on [them]” as a stressor in their life. One participant reported responding with a sense of humor when peers ask how to say “hi” in Chinese: “Mostly, when I tell them, they ask me, ‘how do you say “hi” in Chinese?’ And I’m like, ‘konnichi wa’ [which means “hello” in Japanese][laughs].”

#### Parent

Although parent participants generally denied significant social stressors for their children, about half of the sample endorsed *social skills challenges* such as emotional dysregulation, social skills deficits, or social immaturity. One parent noted her child “is struggling with her friends, just trying to find her place.” Another parent reported her child does not understand sarcasm. Participants often noted sensitivity in their children, which was often described to be both a strength and something that could contribute to interpersonal challenges. Finally*,* speech and hearing were noted to increase the risk of social challenges. This was evident in the overlap of families who endorsed both speech/hearing impairment as well as social/emotional challenges. One parent described both speech and social skills challenges as a source of bullying:

… some of that might be brought upon by behaviors too. You know, when you react silly, then kids are more likely to kind of use you as a target. So, um, [*sic*] but speech as well. I don't, I haven't heard anything, but I still wonder if you know, if there's some of that.

Another participant indicated she is “probably more concerned about how people think about how she talks than about how she looks or her color.” One mother pointed out her child felt singled out for being removed from class to receive speech therapy. Another parent reported the hearing impairment was an unexpected outcome following adoption.

## Discussion

### Factors Contributing to Positive Self-Concept

Biopsychosocial (Engel, 1977) and ecological (Brofenbrenner, 1979, 1986) frameworks are helpful for contextualizing results. In particular, the interpersonal dynamics between children and other individuals, groups, communities, and systems offer a valuable means for discussing the cultural considerations of self-concept of the present sample. Integration and application of these theories for health populations have been suggested in previous research (Lehman et al., 2017).

#### Biological

The superordinate theme of *coping with cleft* for child and parent subsamples characterizes the helpful strategies employed by children and their parents to cope with CLP. Child participants communicated they were accustomed to CLP as part of their lived experience, having never known anything else. When asked to describe CLP, child participants typically had difficulty finding the words to accurately explain it. For those who articulated a reply, they used simple language indicative of a *concrete understanding of CLP*, likely reflective of both cognitive development and potential self-consciousness or hesitance to discuss the topic. Surgeries were emphasized as something associated with CLP, with child participants often remembering bone graft surgery occurring within the last 1 to 2 years. In coping with surgeries, a consistent theme was that family supported them prior to and following surgery. It seemed child participants had internalized messages of safety, lack of pain, and positive outcomes associated with surgery. Although CLP itself was not a salient factor contributing to self-concept, for those with speech and/or hearing difficulties, CLP played a more active role. Speech and hearing impairments were associated with a sense of feeling different than peers and a greater likelihood of interpersonal difficulties.

#### Psychological

Psychological factors contributing to positive self-concept identified by the sample included pride and self-efficacy in activities, endorsement of positive attributes, identification with individual and family values, and appearance perceptions. Children endorsed positive attributes, often suggestive of the values they had internalized thus far in their lives. For example, children are identified as being kind, helpful, or creative.

#### Social

The family was the most salient social factor contributing to positive self-concept. Children communicated a sense of satisfaction with their place in the family. Child participants described feeling supported by parents in the context of peer, school, and medical difficulties. Status as an adopted child also appeared to support children's sense of belonging in the family. Parent participants consistently described a cohesive, supportive family system that emphasized belonging and togetherness. All of the families included 2 parents, which could be 1 structural element supporting togetherness.

Faith and/or religion were identified by many of the parent participants as an important family value and source of support. Because international adoption agencies are often faith-based, it is possible that for this population, religious affiliation is more common than in the general population. Religious affiliation has been identified as a coping tool for stress in previous research (Ano and Vasconcelles, 2004), though there is a paucity of research that examines religious coping for a child or CL/P populations.

In addition to the family, peer relationships were identified as social factors contributing to self-concept. Both positive and negative peer interactions were described by the present sample. On the one hand, it can be inferred child participants benefitted from peer interactions in the course of activity involvement and school. On the other hand, over half of the present sample endorsed a degree of interpersonal difficulty. Interpreting peer questions as curiosity, without malicious intent, was identified as an effective coping strategy. Parents appeared to provide significant support in talking through interpersonal situations and reframing negative peer experiences. This has been implicated in previous literature (see Chapados, 2000) as helpful for coping with bullying and teasing.

### Cultural Identity as Part of Self-Concept

Child participants expressed pride in their adoption identity. Adoption books, binders, and photos allowed children to understand their adoption story in a meaningful way. Child participants did not emphasize their race or Chinese heritage as part of their self-concept. Because child participants generally did not have first-hand memory of China, this may indicate the inherent difficulty of connecting to a culture to which the children and parents both have limited direct knowledge. Participants incorporated Chinese culture into their lives through tangible items, holiday observations, and food. Some participants discussed talking openly to their children about race. Families did not endorse immersive cultural experiences, such as taking language classes or regularly attending cultural events. Parents described cultural exposures to be child-led and indicated engagement in these activities tended to diminish over time with parents often citing ambivalence on the part of their child(ren).

Literature regarding transracial adoption can help contextualize cultural themes of the present results. Transracial adoption has a history of controversy in the United States since the trend began in the 1950s to 1960s (Barn, 2013). Adverse effects on cultural identity and belonging have been noted for both domestic and international transracial adoptees (see Feigelman, 2000; Samuels, 2009). Indeed, racial/cultural identity and cultural competence of parents have been primary themes in transracial adoption literature (Barn, 2013). Despite the documented concerns, there is also literature in support of transracial adoption suggesting benefits outweigh disadvantages (see Bartholet, 2007; Simon and Roorda, 2009).

Parents are responsible for the “transmission of cultural values, beliefs and behaviors that promote racial/ethnic identity development.” They are also responsible for “helping the child develop appropriate strategies to adequately confront prejudice, racism and discrimination” (Barn, 2013). Findings from 1 systemic review found that “frequency of cultural experiences, preparation for bias, [and] parent and family discussion of differences in race” (p. 456) was associated with healthy adoptee outcomes (Montgomery and Jordan, 2018). Parent participants in the present study consistently denied their children had experienced racial discrimination. The bullying and teasing endorsed by both subsamples centered around other topics including speech, body size, facial appearance, and behavior. It is possible these accounts may accurately reflect a lack of discrimination. It is also possible children and their parents could be unaware of the most common form of discrimination, microaggressions, which are subtle remarks that tend to invalidate, insult, or otherwise denigrate an individual based on group membership (Sue, 2010). It is important to note that data for the present study were collected prior to the COVID-19 pandemic and rise in Asian American discrimination and hate crimes (Pew Research Center, 2021); the experiences of race-related bullying/discrimination in children adopted from China may have changed in the last 2 years.

Few studies have examined experiences of microaggressions in childhood (Kohli et al., 2018); thus, little is known about how racial microaggressions during school-age years impact self-concept later in life. However, there is research documenting longitudinal negative effects of microaggressions on k-12 academic outcomes (Compton-Lilly, 2019) and lower self-esteem in college students (Nadal et al., 2014). Additional research has described the deleterious impact of racism-related stress for Asian-Americans (see Hwang and Goto, 2008; Liang and Fassinger, 2008). Thus, although participants in this sample denied race-related bullying and discrimination, and did not emphasize race/ethnicity as part of their cultural identity or self-concept, the aforementioned literature suggests parents can benefit from education on racial/ethnic socialization. Further, while interpreting questions about CL/P, adoption, or race benignly may be protective, it is possible such a coping skill does not capture the nuance that may exist for racial minority children.

### Implications for Providers

#### Medical providers

Participants in the present sample endorsed overall adaptive coping with frequent medical appointments, surgeries, and ongoing therapies (eg, speech). However, bone graft surgery was a salient recent challenge for families. It is recommended providers give families education on surgery aftercare including expectations around appearance changes and wound dressing in advance of the surgical procedure. Psychoeducation on pediatric medical traumatic stress and secondary medical traumatic stress may also be beneficial and could be given by medical or psychology providers.

Some of the families in the present sample reported having consulted with medical teams prior to the adoption, once they had learned of their child's CLP diagnosis. Families also reported that although adoption agencies were typically thorough in preparing families for the adoption process, complete information about CLP was not always available. In particular, complete information about the severity of their child's CLP, nature of previous medical care, and likely medical needs upon entering the United States was not always clearly delineated. Medical providers working with these families during the preadoption phase can offer consultation to educate and prepare parents for the range of possible CL/P sequelae.

Finally, in light of present findings implicating the benefit of social support for these families, it is recommended medical teams facilitate communication between families who have adopted children from China. This would allow parents the opportunity to give and receive support around issues that may be unique to their children (eg, discussing adoption, handling regressive or attachment concerns). It would also allow children the opportunity to spend time with others like them, which could normalize their race and adoption experiences, and provide cultural connection.

#### Mental health providers

Findings from the present study support the presence of or access to a psychologist and social worker, as per American Cleft Palate-Craniofacial Association Standards for Team Care (ACPA, 2019). While embedded mental health providers are likely most beneficial to provide immediate consultation and intervention for patients and their families, it is recognized this is not always possible. The present sample had a higher rate of social and/or emotional problems compared to peers without CL/P, with about half of the sample reporting a degree of concern in these domains. However, children were not assessed for a current diagnostic and statistical manual of mental disorders, fifth edition diagnosis, so it is unknown what portion of the present sample met the criteria for a clinically significant mental health disorder. Nevertheless, the present findings support recommendations from the previous literature (Feragen and Stock, 2016) to screen children with CL/P for social/emotional concerns. It should be also noted that the present sample described greater social challenges when there was greater impairment in speech and when there was an associated hearing concern. Thus, mental health providers should be aware of the higher risk for this subset of the population. Social/emotional screening can be conducted by medical providers when necessary; however, screening by the team psychologist or other mental health provider would allow for further assessment and tailored referrals when pertinent.

Regarding interventions to support social functioning, mental health providers can assess child and family attitudes to peer questions, teasing, bullying, or discrimination. It may be a helpful coping strategy to reframe peer questions as a normative curiosity rather than maliciousness as appropriate. Mental health providers working with families can role play with children to support the practice of responses to peer questions or comments, or they can provide instruction to parents for social rehearsal.

Finally, mental health providers can give parents education and resources regarding racial and ethnic socialization, identity development, and discrimination. Parents may also benefit from learning about how racial identity may change with time and development. Open and honest conversations about CL/P, adoption, and race should be encouraged to normalize stigmatized identities. Psychologists or other mental health providers may be able to provide education to parents on microaggressions and types of discrimination (eg, individual, institutional, and structural) their children may face currently or in the future. Strategies for assisting children through general social problems would also be applicable in this respect. Parents can support their child's coping with instances of racial discrimination by fostering open dialogue around race and other individual differences and social rehearsal.

### Directions for Future Research

Much of the literature has examined pediatric craniofacial populations through a deficit lens and thus, future research would benefit from incorporating a focus on strengths and resiliencies (Feragen et al., 2009; Stock and Feragen, 2016). More psychosocial research is needed with children with CL/P adopted from China, as there is little current understanding of how psychosocial outcomes may differ between such individuals and the general pediatric CL/P population. This has meant a lack of research that examines cultural considerations for this specific population.

Future research could also include the application of theoretical models related to cultural and racial socialization practices among international transracial adoptive parents (see Lee et al., 2015). It will be beneficial to also identify samples with medical conditions to explore the potential interaction of cultural and medical factors that contribute to self-concept. Longitudinal studies would be particularly helpful in evaluating self-concept and racial/ethnic identity development over time. Given the preponderance of studies that center on parent perspectives, it is recommended that both future qualitative and quantitative studies aim to emphasize children's experiences. For studies that include parent perspectives, it is recommended fathers be included as most previous studies related to parents' experiences of raising a child with CL/P mainly include mothers (Nelson et al., 2011).

### Strengths and Limitations

Given the lack of previous psychosocial literature with children with CL/P adopted from China, the present study offers valuable considerations that can guide future research and afford medical and mental health providers’ further understanding of this population. An additional strength of the present study is the inclusion of both child and parent data. This allowed for additional perspectives that could not be gained from either subsample alone. Similarly, the focus on school-age children adopted from China allowed for the exploration of lived experiences of an understudied group within the pediatric craniofacial population.

As with all qualitative studies, the ability to generalize the present results to the greater population of children with CL/P adopted from China is limited. Furthermore, it is recommended that current findings be interpreted within the cultural contexts associated with international adoption from China to the United States by predominantly White families. An understanding of the factors that support self-concept in other cultural contexts, such as in different countries or in cases of domestic adoption, may differ given the influence of unique cultural variables. A potential limitation of the present sample was limited diversity for some facets of identity. This included the participation of predominantly mothers and White parents. Although these parent characteristics were homogenous in the parent subsample, the latter characteristics are likely representative of the US population of internationally adopting parents (Hellerstedt et al., 2008). The sample was also geographically restricted to the Midwest; however, the sample represented a mix of rural, suburban, and urban areas.

## Conclusions

Findings from this sample suggested that positive self-concept was supported by pride and self-efficacy in activities, family support, instilment of family values, strategies for coping with a cleft, family belonging, cultural exposure, and normalization of CLP, and other differences. Medical providers can support patients and families by providing education on surgeries, CL/P sequelae and outcomes, and pediatric medical stress. Mental health providers can screen for social and emotional challenges and provide psychoeducation on racial/ethnic socialization, identity development, and coping.
